# CRISPR/Cas12a with Universal crRNA for Indiscriminate Virus Detection

**DOI:** 10.3390/molecules29246066

**Published:** 2024-12-23

**Authors:** Zhenlin Shang, Sitong Liu, Dongxu Liu, Xiaojing Pei, Shujing Li, Yifan He, Yigang Tong, Guoqi Liu

**Affiliations:** 1 School of Light Industry Science and Engineering, Beijing Technology and Business University, Beijing 100048, China; 2 College of Life Science and Technology, Beijing University of Chemical Technology, Beijing 100029, China; 3 Biotecnovo (Beijing) Co., Ltd., Room 801 Suit C Hengtai Center, Building 3 Gate, 18 North Feng Road, Fengtai District, Beijing 100176, China

**Keywords:** viruses, CRISPR-Cas, universal crRNA, nucleic acid detection

## Abstract

Viruses, known for causing widespread biological harm and even extinction, pose significant challenges to public health. Virus detection is crucial for accurate disease diagnosis and preventing the spread of infections. Recently, the outstanding analytical performance of CRISPR/Cas biosensors has shown great potential and they have been considered as augmenting methods for reverse-transcription polymerase chain reaction (RT-PCR), which was the gold standard for nucleic acid detection. We herein utilized Cas12a with universal CRISPR RNA (crRNA) for indiscriminate virus detection by attaching the target to a longer track strand for isothermal amplification. The amplified products contain a domain that is recognized by the Cas12a/crRNA complex, triggering the cleavage of surrounding reporters to produce signals, thereby escaping the target dependence of crRNA recognition. The proposed method allows the same crRNA to detect multiple viral nucleic acids with high sensitivity, including but not limited to *SARS-CoV-2*, human papillomaviruses (*HPV*), *HCOV-NL63*, *HCOV-HKU1*, and miRNA biomarkers. Taking *SARS-CoV-2* and *HPV16* pseudoviruses as examples, this method was proved as a versatile and sensitive platform for molecular diagnostic applications.

## 1. Introduction

The pervasive spread of viruses transcends specific geographical boundaries and climatic conditions, enabling them to affect populations across the globe regardless of location [1]. For example, the *SARS-CoV-2* outbreak and the emergence of multiple variants posed considerable challenges in controlling the pandemic [2,3]. Additionally, various coronaviruses, human papillomaviruses (*HPVs*), and other viruses have posed substantial threats to public health around the world [4,5,6,7]. The nucleic acids extracted from viruses unlock a treasure trove of critical information, offering a pathway to clinical diagnoses that are both sensitive and specific exceptionally [8,9]. Nucleic acid-based molecular diagnostics are essential for pathogenic microorganisms, gene mutations linked to cancer prognosis, and numerous biomarkers indicative of serious diseases [10,11]. Widely utilized nucleic acid detection techniques, including next-generation sequencing and reverse-transcription polymerase chain reaction (RT-PCR), possess the theoretical capability to identify and quantify a variety of nucleic acid targets across numerous applications [12,13]. The development of rapid, cost-effective, and sensitive molecular diagnostic tests is crucial for wide and rapid detection of viruses [14].

Clustered regularly interspaced short palindromic repeats (CRISPR) and CRISPR-associated (Cas) systems are acquired immune systems found in most bacteria and archaea [15,16]. Cas proteins, including Cas12a, Cas13a, and Cas14a, demonstrate both cis and trans-cleavage activities upon binding to their targets. This unique functionality has been extensively harnessed to develop highly sensitive and specific molecular diagnostics methods [17,18] and biosensors [19,20,21,22,23,24]. The great analytical capabilities of CRISPR/Cas have demonstrated remarkable potential and have been considered as augmenting detection methods for RT-PCR [25,26]. Cas12a, belonging to the class 2 type V system, can specifically recognize DNA targets under the guidance of CRISPR RNA (crRNA). A single 42–44 nucleotide (nt) crRNA with an approximate 19 nt direct repeat sequence followed by 23–25 nt of spacer sequences has been hybridized with foreign nucleic acids for Cas12a activation to catalyze the untargeted trans-cleavage of single-stranded DNA (ssDNA) substrates [27,28]. Many studies have indicated that modifications of crRNA can significantly enhance the analytical capabilities of CRISPR/Cas systems [29,30,31]. For instance, the multi-crRNA strategy used to simultaneously identify multiple areas of a target gene enables highly sensitive assay [32,33,34]. Despite the favorable features, there are still some limitations. On the one hand, crRNA lacks versatility and each target sequence requires a new specific crRNA for detection, which significantly increases the difficulty and cost of extensive virus screening. It is well established that crRNA is extremely unstable in the environment and synthesizing crRNAs is both expensive and time-consuming [35,36]. On the other hand, the most widely used Cas12a-based assay is limited to DNA targets, leading to compromised applications in nucleic acid analysis such as RNA targets, so other effectors for Cas13a are required. therefore, it is imperative to design a universal CRISPR/Cas12a detection method for indiscriminate virus detection.

In this study, we developed a strategy employing Cas12a and a universal crRNA for the detection of both DNA and RNA targets, which only requires the binding of short target sequences to an elongated track strand. The isothermal amplification is performed to produce double-stranded nucleic acid products. The amplified products contain a binding region for the universal crRNA, which is then recognized by the Cas12a/crRNA complex, triggering the indiscriminate cleavage of fluorophore-quencher reporters and generating detectable signals. Utilizing this single crRNA approach, we can detect multiple viral nucleic acids with high sensitivity, including but not limited to *SARS-CoV-2*, *HPV*, *HCoV-NL63*, *HCoV-HKU1*, and miRNA biomarkers. *SARS-CoV-2* and *HPV16* pseudoviruses were used as model examples for real sample detection verification. This method is anticipated to offer a universal and sensitive platform for molecular diagnostics.

## 2. Results and Discussion

### 2.1. Working Principle and Feasibility Study

**Figure 1:**
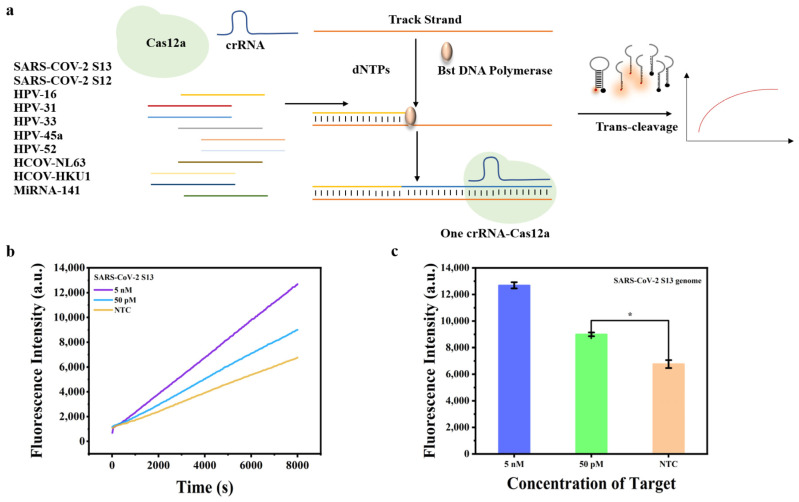
(**a**) The assay workflow CRISPR/Cas12a with universal crRNA for indiscriminate nucleic acid detection; (**b**) Real-time fluorescence intensity of the *SARS-CoV-2 S13* genome using universal crRNA detection; (**c**) fluorescence intensity at 8000 s of *SARS-CoV-2 S13* genome using universal crRNA detection, where *n* = 3 replicates and bars represent mean ± S.D. * *p* < 0.05.

The inevitability of matching a unique crRNA to each target substantially elevates the complexity and expense of CRISPR/Cas-based biosensors. A universal crRNA that can identify various viruses, streamlining nucleic acid analysis while reducing costs, is urgently needed. Figure 1a illustrates the typical assay workflow of CRISPR/Cas12a with universal crRNA for indiscriminate nucleic acid detection. The track combined with various targets was ingeniously designed comprising two key regions: a target binding region located at the 3′ end and crRNA recognition region in the middle of the track. The target hybridized with the track after annealing. Subsequently, the Bst polymerase and dNTPs are introduced to initiate isothermal amplification resulting in elongation of the short target strand and formation of a long double-stranded nucleic acid product. This elongated region of the double-stranded nucleic acid product can be recognized by the Cas12a/crRNA complex, thereby triggering the catalytic activity of Cas12a for collateral cleavage of adjacent reporters to produce signals. Using one crRNA, multiple viral nucleic acids can be detected with high sensitivity, including but not limited to *SARS-CoV-2*, *HPV*, *HCOV-NL63*, *HCOV-HKU1* and miRNA biomarkers, which are expected to provide a universal and sensitive sensing platform for molecular diagnosis applications. We first assessed the feasibility of the proposed method using the S13 genome fragment of *SARS-CoV-2* as the model target. As shown in Figure 1b, the fluorescence intensity gradually increased with concentration variation, which was positive with the target concentration. A sensitivity as low as 50 pM was successfully achieved without any optimization procedures (Figure 1c). The results demonstrated the feasibility of the proposed method.

### 2.2. Experimental Optimization

**Figure 2:**
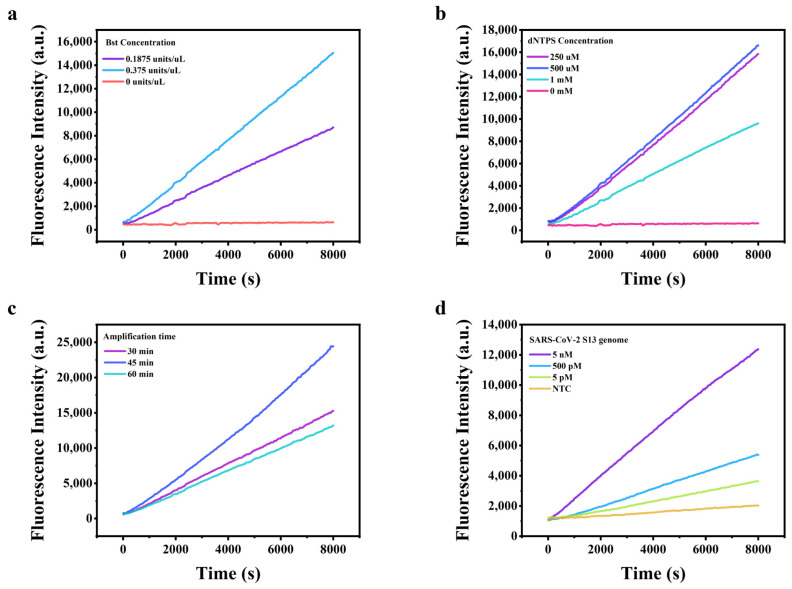
(**a**) Real-time fluorescence values of different Bst DNA polymerases; (**b**) different dNTP mixtures; (**c**) different amplification times; (**d**) real-time fluorescence values of the *SARS-CoV-2-S13* genome fragment detection using optimized conditions.

We further assessed the key factors potentially affecting analytical performance using the S13 genome fragment of *SARS-CoV-2* as the model target. In our previous work, we systematically optimized the CRISPR/Cas12a biosensors, including the concentration of probes and the ratio of crRNA to Cas12a. In addition, we found that the isothermal amplification step critically influenced the sensitivity. Therefore, we optimized the amplification efficiency by adjusting the concentrations of Bst DNA polymerase and dNTP mixture as well as the amplification time. We finetuned the concentration of Bst DNA polymerase by reducing it to 0.5 times of the original concentration (0.375 units/μL) while keeping other components unchanged. A significant decrease was observed in fluorescence intensity compared to the original condition (Figure 2a). Therefore, the concentration of Bst DNA polymerase in the system was confirmed as 0.375 units/μL. Similarly, we optimized the concentration of the dNTP mixture. The results revealed that the best optimal concentration of the dNTP mixture was 250 μM. Upon increasing the concentration to 500 μM, the fluorescence intensity showed a slight improvement, but further increments resulted in waste to some extent (Figure 2b). We used the optimized concentrations of Bst DNA polymerase and dNTP mixture, determined the optimal reaction time and found that 45 min yielded the best results, which we designated as the optimal amplification time (Figure 2c). Finally, utilizing the optimized conditions, we retested the *SARS-CoV-2-S13* genome again (Figure 2d) and observed a significant sensitivity increase from 50 pM to 5 pM, compared with that of Figure 1c.

### 2.3. DNA Target Detection

**Figure 3:**
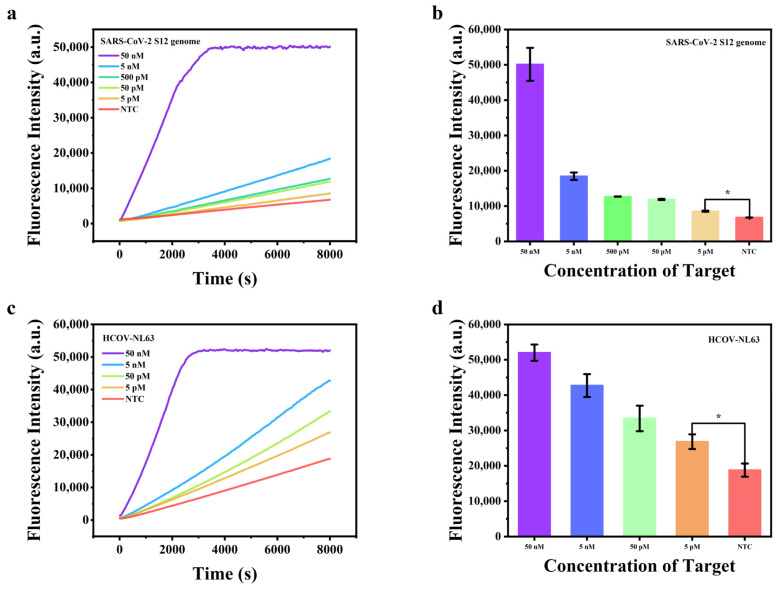
(**a**) Real-time fluorescence intensity with different concentrations of the *SARS-CoV-2 S12* genome; (**b**) fluorescence intensity values with different concentrations at 8000 s of *SARS-CoV-2 S12* genome; (**c**) real-time fluorescence intensity with different concentrations of *HCOV-NL63*; (**d**) fluorescence intensity values with different concentrations at 8000 s of *HCOV-NL63*. *n* = 3 replicates and bars represent mean ± S.D. * *p* < 0.05.

The universal crRNA of the proposed method can detect both DNA and RNA targets. We investigated a variety of targets, with a particular emphasis on *SARS-CoV-2*, the coronavirus, as well as numerous high-risk genotypes of human papillomavirus (HPV) and microRNA (miRNA) in subsequent experiments. With the optimized conditions, we detected the S12 genome fragment specific to *SARS-CoV-2* firstly. A steady rise in fluorescence intensity corresponding to the escalating concentration of the target was observed. Notably, the fluorescence of 5 pM could be distinctly differentiated from the No Template Control (NTC) group (Figure 3a,b). Subsequently, we tested other coronaviruses using the same protocol. When the targets were *HCOV-NL63* (Figure 3c,d) and *HCOV-HKU1* (Figure S1), the system could detect the targets at a minimum concentration of 5 pM. We then employed the proposed method to detect high-risk genotypes of HPV. For HPV-16, HPV-31, HPV-33, and HPV-52 were detected, the system could detect the targets at a minimum concentration of 5 pM (Figures S2–S5). When the target was HPV-45a, the minimum detectable concentration was 50 pM (Figure S6). The decrease in sensitivity may be due to differences in binding efficiency of target-track strand, resulting in different quantities of amplified products stimulating the signal generated by Cas12a.

### 2.4. RNA Target Detection

**Figure 4:**
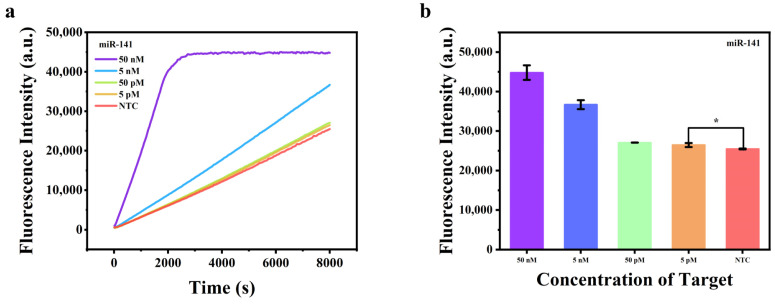
(**a**) Real-time fluorescence intensity of miR-141 with different concentrations; (**b**) fluorescence intensity values at 8000 s of miR141 with different concentrations. *n* = 3 replicates and bars represent mean ± S.D. * *p* < 0.05.

We further investigated the feasibility of the proposed method to test RNA targets. MicroRNAs are single-stranded non-coding RNA molecules composed of approximately 18–25 nt, which play a crucial role in the negative regulation of target genes through translation inhibition or mRNA degradation [37]. MicroRNA-141 (miR-141) is of essential importance in the regulation of cancer occurrence and progression [38]. We chose miR-141 as model RNA targets in this section. The miRNA triggers a polymerization reaction from the 3′-end to synthesize the double-stranded nucleic acid product in the presence of polymerase and dNTPs [39]. We investigated two commonly used polymerases, phi29 and Bst DNA polymerase, for isothermal amplification and found that both could effectively amplify miR-141 (Figure S7). To ensure the integrity of DNA detection, we chose Bst DNA polymerase for subsequent concentration gradient experiments. Specifically, as the miR-141 concentration increased, the fluorescence intensity increased accordingly and a value as low as 5 pM was achieved successfully (Figure 4a,b). All the above results showed that the proposed method was capable of detecting both DNA and RNA targets.

### 2.5. Real Sample Detection

**Figure 5:**
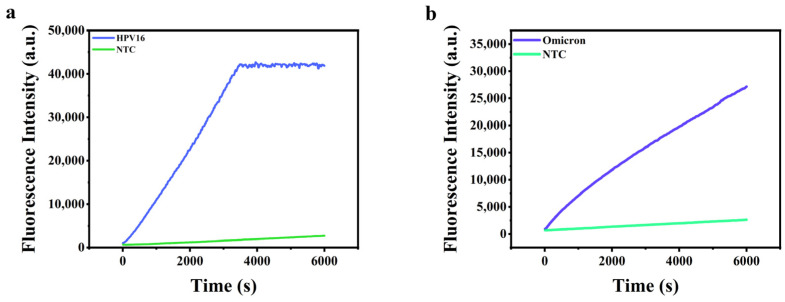
(**a**) Real-time fluorescence intensity of the HPV-16 pseudovirus; (**b**) real-time fluorescence intensity of the Omicron pseudovirus.

Encouraged by the above results, we proceeded to investigate the feasibility of the proposed method to detect real samples. We obtained pseudoviruses of HPV-16 and Omicron of *SARS-COV-2* as model targets. It is worth noting that the pseudoviruses were non-transmissible to ensure safe handling of operators. PCR and RT-PCR amplification were used with Lambda exonuclease to convert the resulting dsDNA amplicon into ssDNA, respectively. The ssDNA target was hybridized with beforehand track stands to form a two-dimensional structure. Bst DNA polymerase was then employed for amplification at 37 °C for 45 min, and the resulting dsDNA was detected with Cas12a/crRNA. As shown in Figure 5, the fluorescence signals of both HPV-16 and Omicron of *SARS-COV-2* pseudovirus were significantly higher than that of the amplification control, demonstrating the successful applications of the proposed method for the detection of pseudoviruses in real samples.

## 3. Materials and Methods

### 3.1. Materials and Reagents

All synthetic nucleic acid oligonucleotides listed in Tables S1 and S2 and diethylpyrocarbonate (DEPC)-treated water were purchased from Sangon Biotech Co., Ltd. (Shanghai, China). Lba Cas12a, Buffer 3.1, Buffer 2, Bst DNA Polymerase, dNTP and Q5 High-Fidelity DNA Polymerases were purchased from New England Biolabs (Beijing, China). RNase inhibitors were purchased from Takara Biotechnology Co., Ltd. (Dalian, China). An RNase-free environment was used throughout the experiments with DEPC-treated water and RNase-free tips and tubes.

### 3.2. Detection of S13 SARS-CoV-2 Using Universal crRNA

First, 1 μL of 1 μM DNA template track strand, 1 μL of *SARS-CoV-2 S13* target, and 5.625 μL of Buffer 2 were added to a centrifuge tube and subjected to annealing operation, and the secondary structure was formed by cooling down the temperature from 95 °C to 25 °C. Subsequently, 0.375 μL of 8000 units/mL Bst DNA polymerase (0.1875 units/μL) and 2 μL of 10 mM dNTP solution (500 μM) were added, and the reaction was carried out at 37 °C for 45 min, followed by warming to 85 °C in 10 min for the inactivation of enzymes and cooling to room temperature. Finally, 0.5 μL of 20 μM crRNA, 0.5 μL of 10 μM Cas12a protein, 7.5 μL of Buffer 3.1, 0.5 μL of RNAase inhibitor, and 1 μL of 10 μM molecular beacon reporter (500 nM) were added, and the resulting mixture was put into a real-time fluorescence PCR instrument (Hangzhou Bio-Gener Technology Co., Ltd., Hangzhou, China) and kept at 47 °C for 8000 s to collect fluorescence signals.

### 3.3. Experimental Optimization

#### 3.3.1. Optimization of Bst DNA Polymerase Concentration

Next, 1 μL of 1 μM DNA template track strand (100 nM), 1 μL of different concentrations of *SARS-CoV-2-S13* genomic target (5 nM, 500 pM, 50 pM, 5 pM, 0), and different volumes (6.25 μL, 6.5 μL) of Buffer 2 were added to a set of centrifuge tubes for annealing operation, and cooled down from 95 °C to 25 °C to form the secondary structure. Subsequently, different volumes (0.75 μL, 0.5 μL) of 8000 units/mL Bst DNA polymerase (0.375 units/μL, 0.1875 units/μL) and 1 μL of 10 mM dNTP solution (250 μM) were added, respectively. The subsequent procedure is the same as that in Section 3.2.

#### 3.3.2. Optimization of Concentrations of dNTP Mixture

In this stage, 1 μL of 1 μM DNA template track strand (100 nM), 1 μL of *SARS-CoV-2-S13* genomic target (5 nM, 500 pM, 50 pM, 5 pM, 0), and (6.25 μL, 5.25 μL, 3.25 μL) of Buffer 2 were added to a set of centrifuge tubes for annealing operation and cooled down from 95 °C to 25 °C to form the secondary structure. Subsequently, 0.75 μL of 8000 units/mL Bst DNA polymerase (0.375 units/μL) and (1 μL, 2 μL, 4 μL) 10 mM dNTP solution (250 μM, 500 μM, 1 mM) were added, respectively. The subsequent procedure is the same as in Section 3.2.

#### 3.3.3. Optimization of the Amplification Time

In this stage, 1 μL of 1 μM DNA template track strand (100 nM), 1 μL of *SARS-CoV-2-S13* genomic target (5 nM, 500 pM, 50 pM, 5 pM, 0), and 6.25 μL of Buffer 2 were added to a set of centrifuge tubes for annealing operation and cooled down from 95 °C to 25 °C to form the secondary structure. Subsequently, 0.75 μL of 8000 units/mL Bst DNA polymerase (0.375 units/μL) and 2 μL of 10 mM dNTP solution (500 μM) were added, respectively. The remaining procedure is the same as in Section 3.2.

### 3.4. Detection of DNA Target

In this stage, 1 μL of 1 μM DNA template track strand (100 nM), 1 μL of DNA targets (*SARS-CoV-2-S12*, *SARS-CoV-2-S13*, *HPV-16*, *HPV-31*, *HPV-33*, *HPV-45a*, *HPV-52*, *HCOV-NL63*, *HCOV-HKU1*) with different concentrations, and 6.25 μL of Buffer 2 were added to a set of centrifuge tubes for annealing operation and cooled down from 95 °C to 25 °C to form the secondary structure. Subsequently, 0.75 μL of 8000 units/mL Bst DNA polymerase (0.375 units/μL) and 1 μL of 10 mM dNTP solution (250 μM) were added and the reaction was carried out at 37 °C for 45 min, followed by warming up to 85 °C 10 min for inactivation and cooling down to room temperature. Finally, 0.5 μL 20 μM crRNA, 0.5 μL 10 μM Cas12a protein, 7.5 μL Buffer 3.1, 0.5 μL RNAase inhibitor, and 1 μL 10 μM reporter (500 nM) were added, and the resulting mixture was put into a real-time fluorescence PCR instrument and reacted at 47 °C for 8000 s to collect fluorescence signals.

### 3.5. Detection of RNA Target

In this stage, 1 μL of 1 μM DNA template track strand (100 nM), 1 μL of RNA targets (miRNA-141) (50 nM, 5 nM, 500 pM, 50 pM, 5 pM, 0), and 6.25 μL of Buffer 2 were added to a set of centrifuge tubes for annealing operation and cooled down from 95 °C to 25 °C to form the secondary structure. The remaining procedure is the same as in Section 3.4.

### 3.6. Detection of Real Samples

To verify the ability of the proposed method for real sample applications, *SARS-COV-2* and HPV-16 pseudo-virus were selected as model targets for the real sample detection verification. Target fragments were obtained using RT-PCR and PCR amplification and lambda exonuclease function to obtain the ssDNA target, respectively. Specifically, 1 μL of 1 μM track strand (100 nM), 1 μL of the target, and 6.25 μL of Buffer 2 were added to a centrifuge tube for annealing operation, and the secondary structure was formed by cooling down from 95 °C to 25 °C. The remaining procedure is the same as in Section 3.4.

## 4. Conclusions

In summary, we developed a Cas12a and universal crRNA strategy for indiscriminate DNA or RNA target detection that simply requires the binding of short targets to the elongated track strand, followed by isothermal amplification to yield double-stranded nucleic acid products recognized by Cas12a. Using the same crRNA, nucleic acids of various viruses, including but not limited to *SARS-CoV-2*, HPV, *HCOV-NL63*, *HCOV-HKU1*, miRNA biomarkers, etc., were detected with sensitivity as low as 5 pM. With the HPV-16 and *SARS-CoV-2* pseudovirus, the real sample application of the proposed method was achieved. This approach expands the design strategies and is expected to provide a universal and sensitive sensing platform for molecular diagnostics.

## Data Availability

Data are contained within the article and Supplementary Materials.

## Supplementary Materials

The following supporting information can be downloaded at: https://www.mdpi.com/article/10.3390/molecules29246066/s1, Table S1: Nucleic acid sequences used for DNA and RNA virus detection; Table S2: Nucleic acid sequences used in the real sample testing; Figure S1: Real-time fluorescence intensity of the HCOV-HKU1; Figure S2: Real-time fluorescence intensity of the HPV-16; Figure S3: Real-time fluorescence intensity of the HPV-31; Figure S4: Real-time fluorescence intensity of the HPV-33; Figure S5: Real-time fluorescence intensity of the HPV-52; Figure S6: Real-time fluorescence intensity of the HPV-45a; Figure S7: Real-time fluorescence intensity of miRNAs amplified by Phi29 DNA Polymerase and Bst DNA Polymerase.
