# Supplementary material for: CRISPR/Cas12a with Universal crRNA for Indiscriminate Virus Detection
Source: Molecules. 2024 Dec 23;29(24):6066. doi: 10.3390/molecules29246066 (PMC11676733; doi:10.3390/molecules29246066)
Supplement: Supplementary file 1 [file molecules-29-06066-s001.zip › molecules-3342645-supplementary.pdf]

CRISPR /Cas12a with universal crRNA for indiscriminate virus detection

Zhenlin Shang [1], Sitong Liu [1], Dongxu Liu [1], Xiaojing Pei [1,*], Shujing Li [1], Yifan He [1], Yigang Tong [2] and Guoqi Liu [3]

[1]School of Light Industry Science and Engineering, Beijing Technology and Business University, Beijing 100048, China;

[2]College of Life Science and Technology, Beijing University of Chemical Technology, Beijing 100029, China;

[3]Biotecnovo (Beijing) Co., Ltd., Room 801 Suit C Hengtai Center, Building 3 Gate, 18 North Feng Road, Fengtai District, Beijing 100176, China;

*Correspondence: pxj@btbu.edu.cn

Table S1 Nucleic acid sequences used for DNA and RNA virus detection.

Name	Sequence 5'-3'
Universal crRNA	UAA UUU CUA CUC UUG UAG AU G UAA CUA GCA AGA AUA CCA C
Reporter	6-Texas Red- TGG GAT ATC TTT AAT TTT ATT TTA ACA AGA TAT CCC A-BHQ2-
SARS-CoV-2-S13-target	AACGTGAGTC TTGTAAAACC TTCT
SARS-CoV-2-S12-target	ACTCATTCGTTTCGGAAGAGACAG
HPV-16-target	CTGTCCCAGTATCTAAGGTTGTAA
HPV-31-target	CTGTCCCAGTGTCTAAAGTTGTAA
HPV-33-target	CTTGAAATAGGTAGAGGGCAGCCA
HPV-45a-target	CCCTTCTCCCAGTGGCTCTATTAT
HPV-52-target	CCTGTCTCTAAGGTTGTAAGCACT
HCOV-NL63-target	CAAGTTGGTTGTTTGTTTTGGATT
HCOV-HKU1-target	CCCAATCATCTGGTGTTATTCCTG
MiRNA-141-target	UAACACUGUCUGGUAAAGAUGG
SARS-CoV-2-S13-track	TTG GGT ATA AAA GAT CCT ATT CTC TAT GTG TTA ACA CAT GAT GTT TGT CAA GTT TGT GGA TTT TGG AGA GAT GGC AGT TGT TCC TGT GTA GGT TCA GGT GTC GCT GT
SARS-CoV-2-S12-track	GGT ATA AAA GAT CCT GTG TCT TAT GTT TTG ACA CAT GAT GTT TGT CGA GTT TGT GGA TTT TGG CGG GAT GGA AGT TGT TCA TGT GTT AGC ACT GAC ACT ACT GT
HPV-16-track	GTAAGGATGGCTAGTGTAACTAGCAAGAATACCACGAAAGCAAGAAAAAGAAGTACGCTATTAACTATTAACGTACTTACAACCTTAGATACTGGGACAG

HPV-31-track	GTAAGGATGGCTAGTGTAACTAGCAAGAATACCACG<br>AAAGCAAGAAAAAGAAGTACGCTATTAACTATTAAC<br>GTACTTACAACTTTAGACACTGGGACAG
HPV-33-track	GTAAGGATGGCTAGTGTAACTAGCAAGAATACCACG<br>AAAGCAAGAAAAAGAAGTACGCTATTAACTATTAAC<br>GTACTGGCTGCCCTCTACCTATTTCAAG
HPV-45a-track	GTAAGGATGGCTAGTGTAACTAGCAAGAATACCACG<br>AAAGCAAGAAAAAGAAGTACGCTATTAACTATTAAC<br>GTACATAATAGAGCCACTGGGAGAAGGG
HPV-52-track	GTAAGGATGGCTAGTGTAACTAGCAAGAATACCACG<br>AAAGCAAGAAAAAGAAGTACGCTATTAACTATTAAC<br>GTACAGTGCTTACAACCTTAGAGACAGG
HCOV-NL63-track	GTAAGGATGGCTAGTGTAACTAGCAAGAATACCACG<br>AAAGCAAGAAAAAGAAGTACGCTATTAACTATTAAC<br>GTACAATCCAAAACAAACAACCAACTTG
HCOV-HKU1-track	GTAAGGATGGCTAGTGTAACTAGCAAGAATACCACG<br>AAAGCAAGAAAAAGAAGTACGCTATTAACTATTAAC<br>GTACCAGGAATAACACCAGATGATTGGG
MiRNA-141-track	GTA AGG ATG GCT AGT GTA ACT AGC AAG AAT<br>ACC ACG AAA GCA AGA AAA AGA AGT ACG CTA<br>TTA ACT ATT AAC GTA C CCA TCT TTA CCA GAC<br>AGT GTT A

Table S2 Nucleic acid sequences used in the real sample testing.

Name	Sequence 5’-3’
HPV-16 target	GTTCCTAAAGTATCAGGATTACAATACAGGGTATTTAG<br>AAT
HPV-16-track	ACATTTACCTGACCCCAATAAGTTTGGTTTTCCTGACAC<br>GTAAGGATGGCTAGTGTAACTAGCAAGAATACCACGA<br>AATCCATCCCGATTCATGTCTGCAACAGATACGAATAA<br>GAAAATGGACTCTCGCGAGAACATTGGTCTCCCTACAG<br>GCATGGGCGACCTGAGTGTCAGGAAAACCAAACTTATT

	GGGGTCAGGTAAATGTATTCTAAATACCCTGTATTGTAATCCTGATACTTTAGGAAC
FP-HPV-16--primer	AAGTATCAGGATTACAATACAGGGT
RP-HPV-16-primer	P-ACCAAACTTATTGGGGTCAGGT
FP-HPV-16--track	GTAAGGATGGCTAGTGTAACTAGCAAGAATAC
RP-HPV-16-track	P-GTTCCTAAAGTATCAGGATTACAATACAGGGTATTTAGA
FP-N501Y--primer	GCACACCTTGTAATGGTGTTGAA
RP-N501Y--primer	P-CACCATTAGTGGGTTGGAAAC
FP- N501Y--track	GTAAGGATGGCTAGTGTAACTAGCAAGAATAC
RP- N501Y-track	P-AGCACACCTTGTAATGGTGTTGAAG

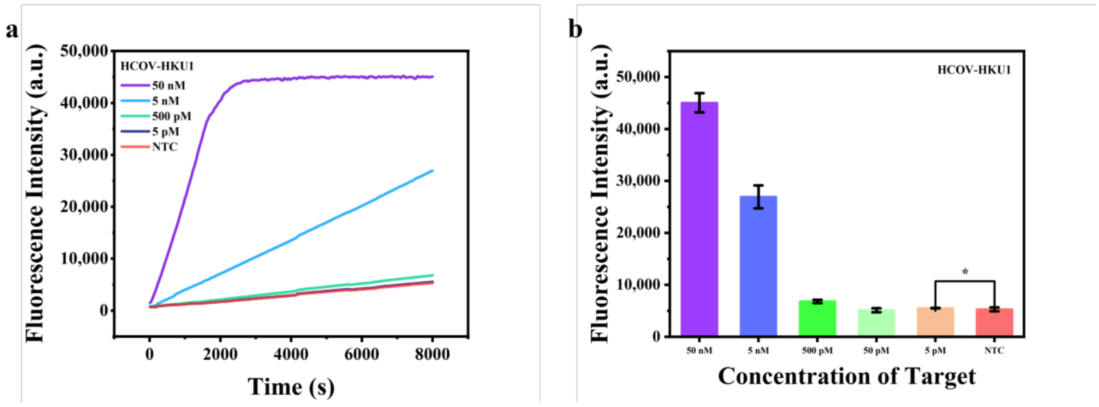

Figure S1 (a) Real-time fluorescence intensity of the HCOV-HKU1. **(b)** Fluorescence intensity values at 8000s of the HCOV- HKU1. *n* = 3 replicates, bars represent mean ± S.D. *P < 0.05.

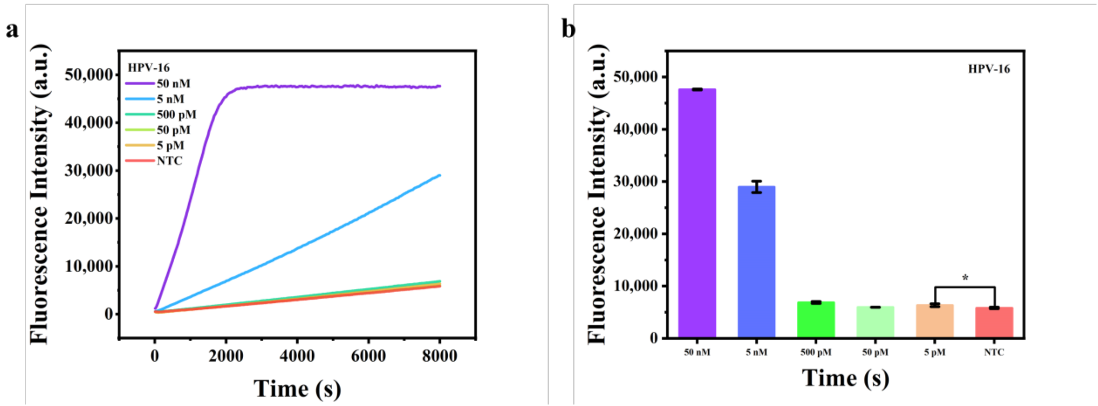

Figure S2 **(a)** Real-time fluorescence intensity of the HPV-16. **(b)** Fluorescence intensity values at 8000s of the HPV-16. *n* = 3 replicates, bars represent mean ± S.D. *P < 0.05.

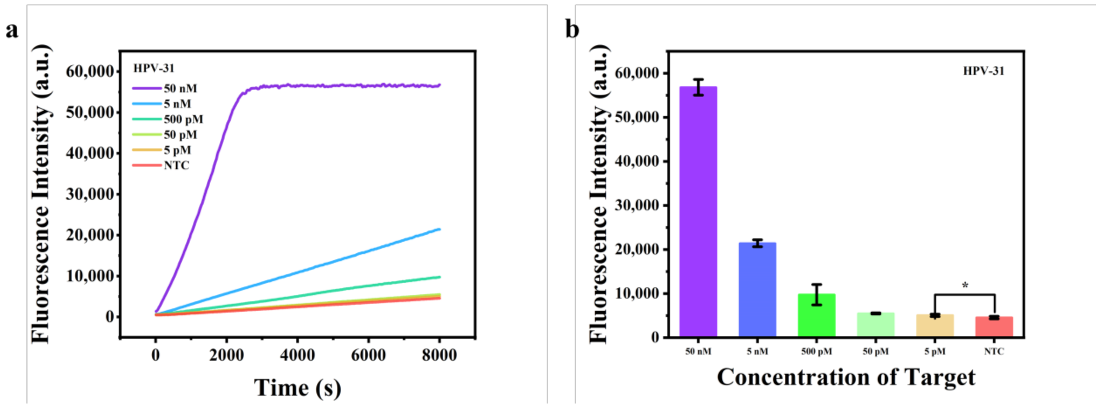

Figure S3 (a) Real-time fluorescence intensity of the HPV-31. **(b)** Fluorescence intensity values at 8000s of the HPV-31. *n* = 3 replicates, bars represent mean ± S.D. *P < 0.05.

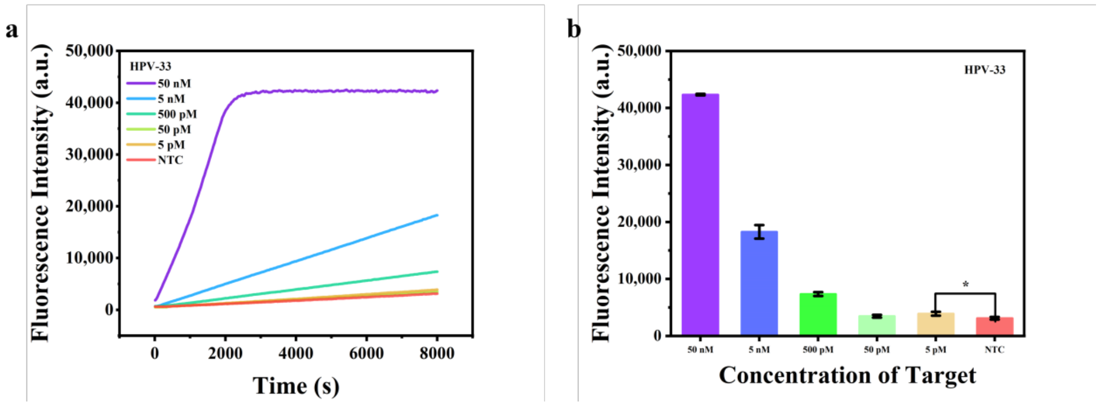

Figure S4 (a) Real-time fluorescence intensity of the HPV-33. **(b)** Fluorescence intensity values at 8000s of the HPV-33. $n = 3$ replicates, bars represent mean ± S.D. *P < 0.05.

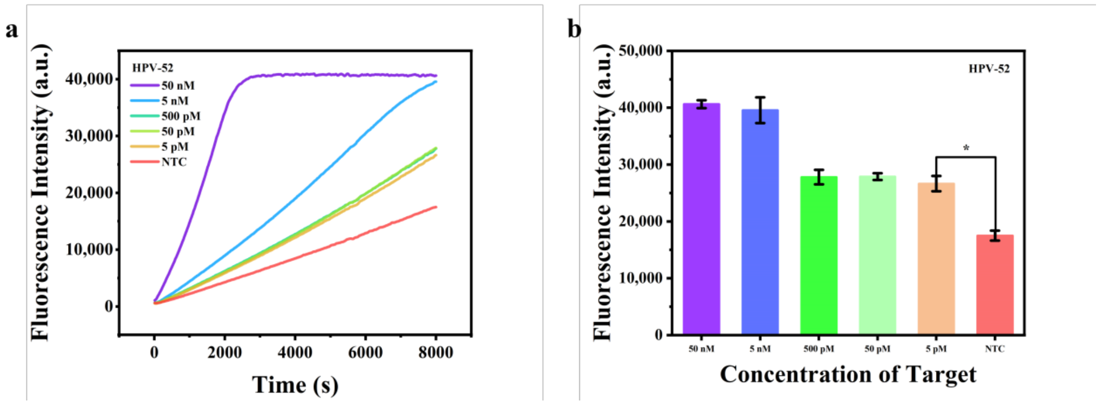

Figure S5 (a) Real-time fluorescence intensity of the HPV-52. **(b)** Fluorescence intensity values at 8000s of the HPV-52. $n = 3$ replicates, bars represent mean ± S.D. *P < 0.05.

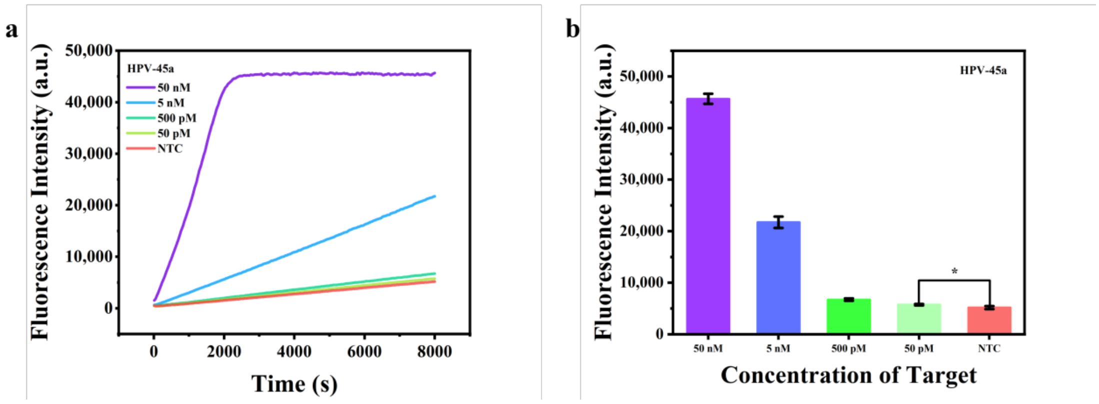

Figure S6 (a) Real-time fluorescence intensity of the HPV-45a. **(b)** Fluorescence intensity values at 8000s of the HPV-45a. $n = 3$ replicates, bars represent mean ± S.D. *P < 0.05.

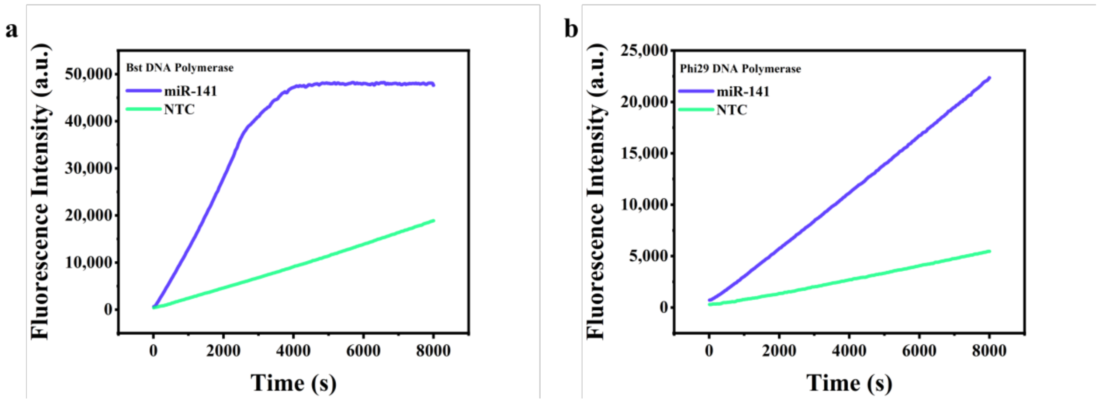

Figure S7 (a) Real-time fluorescence intensity of miRNAs amplified by Phi29 DNA Polymerase and **(b)** Bst DNA Polymerase.
